# Percutaneous Hybrid Approach for an Aortic Paravalvular Leak Associated With a Large Pseudoaneurysm

**DOI:** 10.1016/j.jaccas.2026.107631

**Published:** 2026-04-06

**Authors:** Craig Basman, Perry Wengrofsky, Vladimir Jelnin, Sung-Han Yoon, Mark Anderson, Rachel Spallone, Elie Elmann, George Batsides, Yuriy Dudiy, Ryan Kaple

**Affiliations:** Hackensack University Medical Center, Hackensack, New Jersey, USA

**Keywords:** paravalvular leak, percutaneous transapical, pseudoaneurysm

## Abstract

**Background:**

Closure of complex paravalvular leak (PVL) via percutaneous approach may be challenging.

**Case Summary:**

Our case highlights the rare occurrence of a complex PVL that extended into a pseudoaneurysm with entry sites in the aorta and left ventricular outflow tract far apart. We performed a hybrid percutaneous approach with transapical closure of the left ventricular side and then retrograde transfemoral closure of the aortic side.

**Discussion:**

Patients treated surgically for infective endocarditis may exhibit postoperative changes, including the potential development of PVL. Weakening of the tissue in the tract may lead to aneurysmal growth. Percutaneous single-site exclusion would lead to incomplete treatment; hence, dual-entry closure (aortic and left ventricular outflow tract) is necessary to reduce PVL and prevent aneurysmal expansion.

**Take-Home Messages:**

This case explores the complexities of treating PVL associated with a long and serpiginous tract with aneurysmal growth. Single-site transcatheter exclusion is inadequate to reduce regurgitation and mitigate aneurysmal expansion for serpiginous PVL tracts associated with pseudoaneurysm.

## Visual Summary

**Figure:**
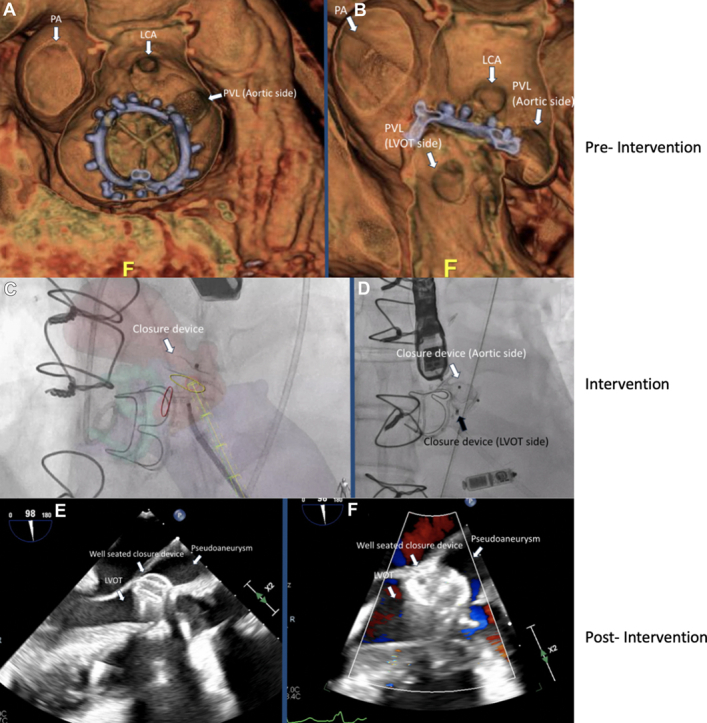

## History of Presentation

A 57-year-old woman with a complicated surgical history presented for follow-up with NYHA functional class II symptoms. She was afebrile with a blood pressure of 120/40 mm Hg and oxygen saturation of 99% on room air. On physical examination, she had a 3/6 diastolic murmur over the right upper sternal border radiating to the apex.

**Take-Home Messages**

• This case explores the complexities of treating PVL associated with a long and serpiginous tract with aneurysmal growth.
• Single-site transcatheter exclusion is inadequate to reduce regurgitation and mitigate aneurysmal expansion for serpiginous PVL tracts associated with pseudoaneurysm.

## Past Medical History

The patient had a known history of bicuspid aortic stenosis after having undergone surgical aortic valve replacement and ascending aortic aneurysm repair in the previous year, complicated by complete heart block requiring dual-chamber pacemaker. She re-presented after 6 months with embolic strokes due to aortic valve and pacemaker lead endocarditis requiring redo surgical aortic valve replacement, lead extraction, and implantation of a leadless pacemaker.

**Visual Summary:**
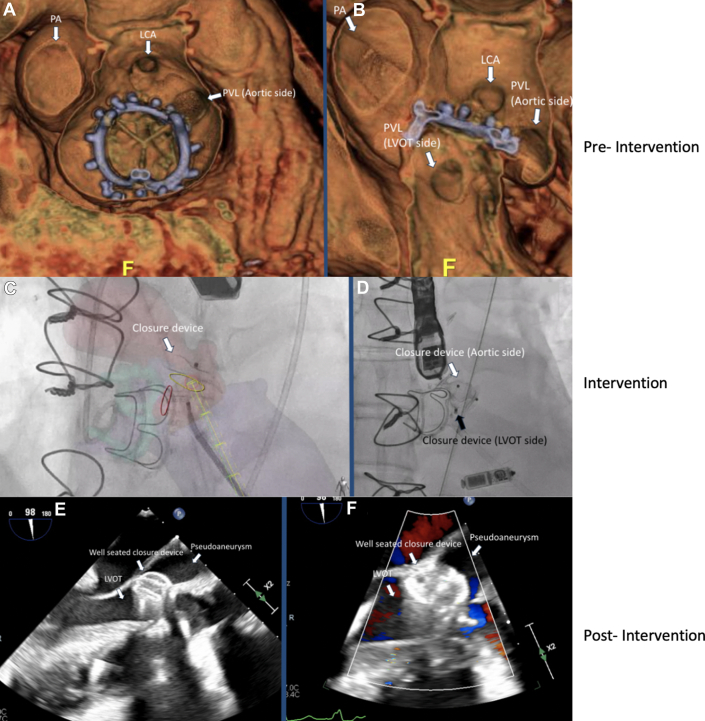
Workup and Treatment of a Large Aortic Paravalvular Leak and Pseudoaneurysm (A and B) Preintervention CT shows the origination of the PVL and the entry into the LVOT. (C) CT-fluoroscopy fusion imaging used to guide the procedure to help close the LVOT side from the transapical approach, and (D) cineangiography showing well-seated closure of the aortic and LVOT side. (E) Postclosure transesophageal echocardiography shows vascular plug preventing flow from the pseudoaneurysm into the LVOT, and (F) color Doppler shows no flow from the pseudoaneurysm into the LVOT. CT = computed tomography; LCA = left main coronary artery; LVOT = left ventricular outflow tract; PA = pseudoaneurysm; PVL = paravalvular leak.

## Differential Diagnosis

The differential diagnosis included bioprosthetic valve failure, congestive heart failure, and paravalvular leak (PVL).

## Investigations

**Figure 1:**
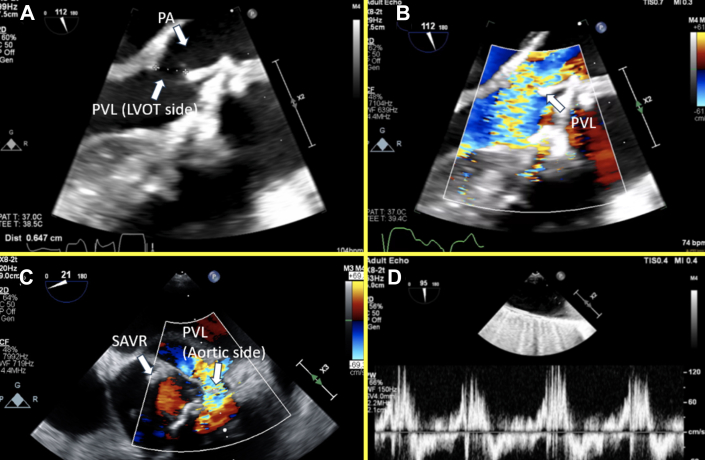
Transesophageal Echocardiography of Paravalvular Leak (A) Transesophageal echocardiography shows PVL originating from a pseudoaneurysm into the LVOT. (B) Color Doppler reveals severe regurgitation through the PVL. (C) The PVL originated above the surgical aortic valve (on the aortic side), and flow wrapped around a serpiginous tract. (D) There was evidence of severe aortic insufficiency, such as holodiastolic reversal. LVOT = left ventricular outflow tract; PVL = paravalvular leak; SAVR = surgical aortic valve.

**Figure 2:**
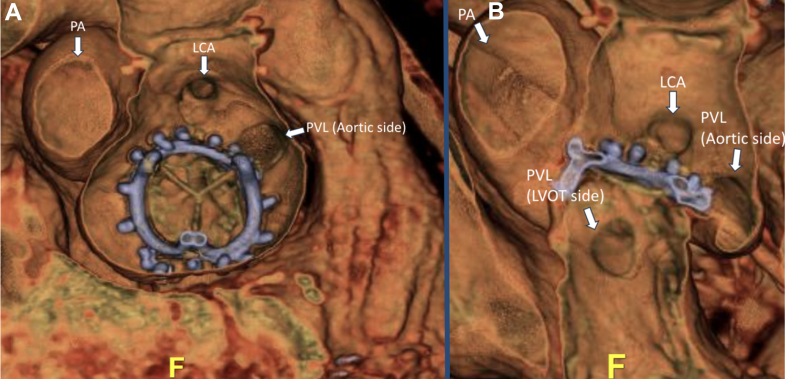
Three-Dimensional Computed Tomography Reconstruction of the Defect (A) From the “surgeon's view,” the PVL is visualized to originate above the surgical aortic valve on the aortic side, adjacent to the left main coronary artery, and a large pseudoaneurysm is visualized near the left/right coronary cusp. (B) A different angle shows the origination of the PVL (aortic side) and the large pseudoaneurysm that re-enters into the LVOT side. CT = computed tomography; LCA = left main coronary artery; LVOT = left ventricular outflow tract; PA = pseudoaneurysm; PVL = paravalvular leak.

The presenting electrocardiogram showed sinus bradycardia. Laboratory testing indicated mild anemia (stable from prior) and normal kidney function with elevated B-type natriuretic peptide of 2,300 pg/mL (normal: <135 pg/mL). Transthoracic echocardiography showed a normal left ventricular function and severe aortic insufficiency (AI) due to PVL. Transesophageal echocardiography (TEE) showed that the PVL originated in the left coronary cusp and communicated with the left ventricular outflow tract (LVOT) by means of long tract involving a pseudoaneurysm around the prior bioprosthetic aortic valve (Figure 1, Video 1). Cardiac computed tomography angiography (CCTA) was obtained, which revealed extensive PVL that originated next to the left main coronary artery (LCA) and extended into a large pseudoaneurysm wrapping around the LVOT and re-entering several millimeters below the surgical valve (Figure 2, Video 2). The CCTA was necessary to identify the entry and exit portions of the defect and analyze in 3 dimensions the tract pathway. The distance of the PVL origin in relation to the LCA was essential for transcatheter planning as well.

## Management

There was discussion within the heart team for a second reoperation within 1 year or to attempt transcatheter closure. We believed the patient to be at high surgical risk, and we therefore attempted a transcatheter approach with the intent to close both the site of origination (aorta) and entry (LVOT).

**Figure 3:**
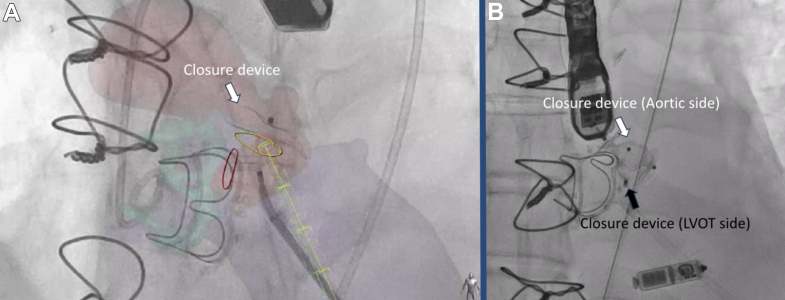
Fluoroscopic Projections of Paravalvular Leak Closure (A) CT-fluoroscopy fusion imaging shows a closure device approaching from the LVOT side. The yellow circle illustrates origination of the PVL on the LVOT side, and the red circle illustrates origination of the PVL on the aortic side. The yellow cylindrical pathway is the anticipated trajectory from the apical site used to guide entry through the PVL into the pseudoaneurysm. (B) Before release of the vascular plug on the aortic side, cineangiography shows both closure devices from the aortic side and the LVOT side well seated. CT = computed tomography; LVOT = left ventricular outflow tract; PVL = paravalvular leak.

**Figure 4:**
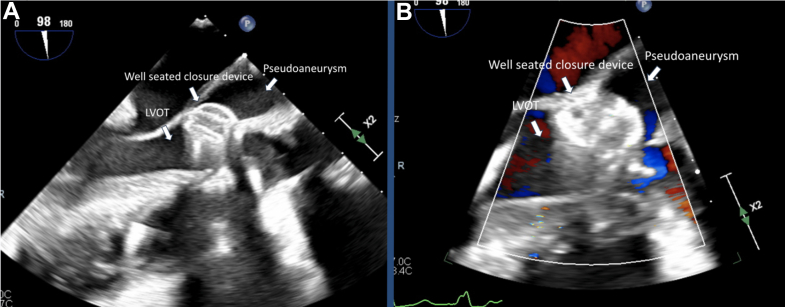
Transesophageal Echocardiography After Paravalvular Leak Closure (A) Transesophageal echocardiography shows the vascular plug preventing flow from the pseudoaneurysm into the LVOT, and (B) color Doppler shows no flow from the pseudoaneurysm into the LVOT. LVOT = left ventricular outflow tract.

**Figure 5:**
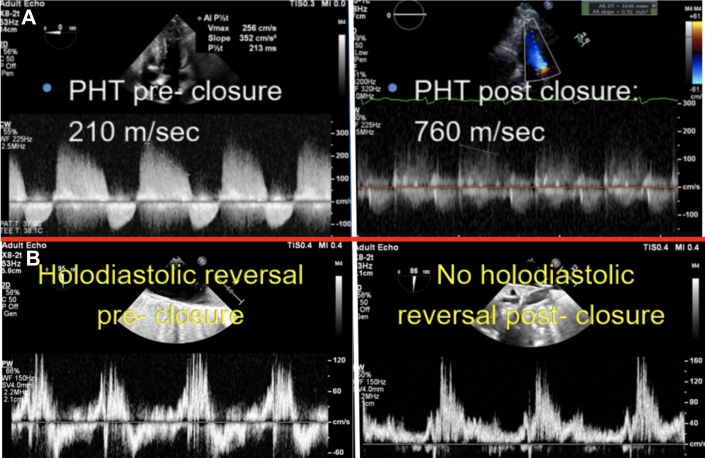
Echocardiographic Markers of Paravalvular Leak Improvement (A) Echocardiographic markers of aortic insufficiency, including pressure half time, improve from preclosure (210 ms) to postclosure (760 ms). (B) Holodiastolic reversal in the aorta is present before closure and normalizes after closure.

The patient was brought to the hybrid operating room, and percutaneous transapical puncture was performed using preoperative CCTA for planning. The puncture site was determined from preprocedure CT (below the 7th rib space) and was performed using transthoracic echocardiography to ensure apical position. Diluted contrast was injected through a micropuncture needle to ensure that we were safely in the LV cavity. Next, the LVOT side was crossed with a 0.014-inch wire (Video 3), and a 7-F slender sheath was advanced across the defect to deploy an 8-mm Amplatzer Atrial Septal Occluder (Abbott) to seal off the LVOT entry site (Figure 3A, Video 4). Next, via transfemoral approach, the aortic entry of the PVL was engaged with a 0.035-inch stiff angled glidewire, and a 7-F shuttle sheath was advanced across her tortuous aorta. A 14-mm Amplatzer Vascular Plug II (Abbott) was deployed, with aortic root angiography confirming a patent LCA (Figure 3B). TEE showed mild turbulent flow around the device with normalization of her holodiastolic reversal and improvement of the pulse pressure by >20 mm Hg (Figures 4 and 5). The transapical puncture site was closed with an 8/6 mm Amplatzer Duct Occluder II (Abbott) (Video 5). A summary of the procedure is available in Video 6.

## Follow-Up

The patient was discharged on postoperative day 1, with improvement of her symptoms at the 1-month follow-up (NYHA functional class I). She was maintained on dual antiplatelet therapy for 3 months (to reduce stroke risk as the devices endothelialized). At 6 months, she continued to report NYHA class I symptoms, and CCTA showed well-seated devices with a mild reduction in pseudoaneurysm size. Given significant aliasing from the multiple devices, the severity of AI was unclear on TTE; hence, magnetic resonance imaging was obtained, which showed mild to moderate residual regurgitation across the aortic valve (regurgitant fraction: 27%, regurgitant volume: 29 mL/beat).

## Discussion

**Figure 6:**
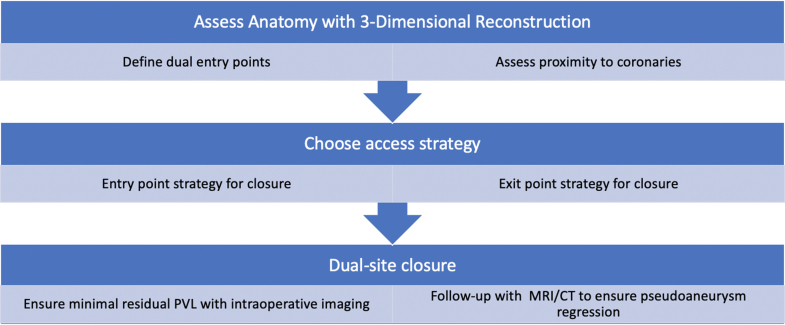
Algorithm for the Approach to Complex Paravalvular Leak With an Aneurysmal Tract CT = computed tomography; MRI = magnetic resonance imaging; PVL = paravalvular leak.

Transcatheter closure for PVL is an option for patients with high surgical risk, typically performed by extending a vascular plug across the lesion to ensure no flow extends around the valve.^1^ However, there are rare cases (typically associated with infection of the prosthesis) whereby the PVL tract extends into aneurysmal tissue.^2^ This pathology causes both AI and puts patients at risk for pseudoaneurysm growth/rupture. Excluding entry from the aortic side would reduce AI, but it would not prevent flow from the LVOT into the defect. A persistent LVOT entrance introduces the risk of the pseudoaneurysm expanding further. Similarly, if isolated LVOT-side closure is performed, flow from the aortic side into the pseudoaneurysm will persist. Hence, in such complex tracts, the LVOT and aortic side both require treatment to prevent pseudoaneurysm growth. An algorithm for the approach to complex PVL with aneurysmal tracts is described in Figure 6.

Surgical correction would be preferred; however, the complications associated with a third sternotomy within 1 year, along with the patient's recent strokes, led the heart team to consider a transcatheter approach for her. The durability of 2 plugs placed in the aortic and LV positions is uncertain, but we felt that should they fail in the future, her surgical risks would be more manageable, having had more time to recuperate from her prior surgeries and stroke.

The LV side had an unfavorable angle for retrograde closure, so the transapical approach was thought to be our best option, with a trajectory directed at the lesion. With standard techniques and the use of preoperative computed tomography to guide entry, percutaneous transapical access can be done safely.^3^ The aortic side was far enough away from the coronary artery and appeared to be a good target for PVL closure. Given the long serpiginous defect, a single plug would not be able to extend across both entrances,^4^ hence, a combination transapical and transfemoral approach was used to achieve adequate closure of both sites.

Multimodality imaging was essential for diagnosis, therapy, and follow-up. CCTA was essential to understand the 3-dimensional spatial anatomy of the defect. TEE was used for intraoperative guidance of the procedure and to understand device stability, and CT-fluoroscopy fusion imaging served as an adjunctive imaging modality to guide entry into the defects. On follow-up, it was difficult to assess AI burden on TEE because of aliasing, so cardiac magnetic resonance imaging was used to assess regurgitation volume. CCTA can also be used at follow-up to assess the extent of pseudoaneurysm regression.

## Conclusions

Our case illustrates a rare complication of infective endocarditis in which a serpiginous PVL associated with a large pseudoaneurysm formed. We used a combination approach of percutaneous transapical and retrograde aortic from the femoral to effectively seal off the orifice of the PVL from the aortic and left ventricular side.

## Funding Support and Author Disclosures

Dr Kaple is a consultant for Abbott and Edwards Lifescience. All other authors have reported that they have no relationships relevant to the contents of this paper to disclose

## Appendix

Video 1 Left Ventricular Outflow View on Transesophageal Echocardiography With Severe Regurgitation From the Pseudoaneurysm Into the LVOT LVOT = left ventricular outflow tract.

Video 2 Three-Dimensional CT Reconstruction Showing the Origination of PVL From the Aortic Side, Wrapping Around the LVOT in a Serpiginous Tract That Forms a Large Pseudoaneurysm and Enters Back Into the LVOT CT = computed tomography; LVOT = left ventricular outflow tract; PVL = paravalvular leak

Video 3 CT-Fluoroscopy Fusion Imaging Showing a Wire Coming Into the Pseudoaneurysm From the Transapical Site The yellow circle illustrates origination of the PVL on the LVOT side, and the red circle illustrates origination of the PVL on the aortic side. The yellow cylindrical pathway is the anticipated trajectory from the apical site used to guide entry through the PVL into the pseudoaneurysm. CT = computed tomography; LVOT = left ventricular outflow tract; PVL = paravalvular leak.

Video 4 CT-Fluoroscopy Fusion Imaging Used to Guide Deployment of the LVOT Plug The yellow circle illustrates origination of the PVL on the LVOT side (which is the one being closed in this video), and the red circle illustrates origination of the PVL on the aortic side. The yellow cylindrical pathway is the anticipated trajectory from the apical site into the pseudoaneurysm. CT = computed tomography; LVOT = left ventricular outflow tract; PVL = paravalvular leak.

Video 5 Cineangiography Showing Both Closure Devices From the Aortic Side and LVOT Side Well Seated With No Excess Motion LVOT = left ventricular outflow tract

Video 6 Summary of the Procedure ADO II = Amplatzer Duct Occluder II; ASD = atrial septal device; AVP II = Amplatzer Vascular Plug II; CT = computed tomography; LVOT = left ventricular outflow tract; PVL = paravalvular leak.

## References

[1] Wells J.A., Condado J.F., Kamioka N. (2017). Outcomes after paravalvular leak closure: transcatheter versus surgical approaches. JACC Cardiovasc Interv.

[2] Ussia G.P., Cammalleri V., De Vico P., Sergi D., Romeo F. (2013). Transcatheter closure of paravalvular leak secondary to left ventricular peri-annular pseudoaneurysm. Eur Heart J Cardiovasc Imaging.

[3] Kliger C., Jelnin V., Sharma S. (2014). CT angiography-fluoroscopy fusion imaging for percutaneous transapical access. JACC Cardiovasc Imaging.

[4] Basman C., Kaple R., Yoon S.H. (2025). Transcatheter closure of a complex paravalvular leak associated with a large ventricular pseudoaneurysm. J Invasive Cardiol.

